# Real-Time Estimation of User Adaptation During Hip Exosuit-Assisted Walking Using Wearable Inertial Measurement Unit Data and Long Short-Term Memory Modeling

**DOI:** 10.3390/biomimetics11020096

**Published:** 2026-02-01

**Authors:** Cheonkyu Park, Alireza Nasizadeh, Kiho Lee, Gyeongmo Kim, Giuk Lee

**Affiliations:** 1 School of Mechanical Engineering, Chung-Ang University, Seoul 06974, Republic of Korea; lionbecky@naver.com (C.P.); alireza@cau.ac.kr (A.N.); dlrlgh1324@cau.ac.kr (K.L.); woogen1206@cau.ac.kr (G.K.); 2 HUROTICS Inc., Seoul 06912, Republic of Korea

**Keywords:** exosuits, gait variability, real-time monitoring, user adaptation, wearable robots

## Abstract

Wearable robots can improve human walking economy; however, their effectiveness depends on user adaptation to assistance. This study introduces a framework for real-time estimation of user adaptation that relies only on wearable sensor data during operation. Metabolic measurements were used solely to establish the ground truth adaptation curves for model training and validation but are not required for real-time inference. Five healthy adults completed six days of treadmill walking while wearing a soft hip exosuit that provided hip extension assistance. Thigh-mounted inertial measurement units recorded step timing and hip-angle trajectories, from which three variability-based features (step-frequency variability, maximum hip-flexion variability, and maximum hip-extension variability) were extracted. A Long Short-Term Memory (LSTM) model used these gait-variability inputs to estimate each user’s adaptation level relative to a metabolic cost benchmark obtained from respiratory gas analysis. Across sessions, the metabolic cost decreased by 9.0 ± 5.6% from Day 1 to Day 6 (*p* < 0.01) with a mean time constant of 202 ± 78 min, In contrast, the variability in step frequency, maximum hip flexion, and maximum hip extension decreased by 66.4 ± 6.8%, 37.9 ± 24.2%, and 42.8 ± 10.6%, respectively, indicating that these reductions were users’ progressive adaptation to the exosuit’s assistance. Under leave-one-subject-out (LOSO) evaluation across five participants, 59.2% of the model predictions fell within ±10 percentage points of the metabolic cost–based adaptation curve. These results suggest that simple kinematic variability measured with wearable sensors can track user adaptation and support practical approaches to real-time monitoring. Such capability can facilitate adaptive control and training protocols that personalize exosuit assistance.

## 1. Introduction

Walking economy is a fundamental determinant of human mobility, endurance, and rehabilitation outcomes. Increased metabolic cost during locomotion can severely limit daily activities and quality of life. To address this, wearable robots have emerged as a promising solution to augment muscular strength and enhance walking efficiency [1]. Since the first demonstration of a 6% improvement in walking economy using an ankle exoskeleton in 2013 [2], numerous devices have been developed to reduce metabolic energy consumption. For instance, soft wearable robots have been shown to reduce metabolic cost by 9.3% during walking and by approximately 4% during running [3]. These findings underscore the potential of wearable robots to alleviate the metabolic burden of locomotion. 

Despite these advances, the reported benefits of wearable robots often vary across users. Maximizing robotic performance requires addressing two distinct but complementary factors: personalized assistance strategies and user adaptation. Personalized assistance tailors the robot’s control parameters to individual biomechanics. Human-in-the-loop optimization approaches have demonstrated substantial benefits, with studies reporting metabolic cost reductions of approximately 5–20% via optimized assistance profiles [4,5,6]. However, optimizing the controller alone is insufficient if the user has not yet learned to utilize the assistance effectively. 

This highlights the second critical factor: user adaptation. Adaptation refers to the user’s physiological and neurological acclimatization to the device over time. Early use often involves unfamiliar or inefficient movements, which gradually improve as users adapt [7]. For example, ankle exoskeleton users showed an approximately 10% reduction in metabolic cost during initial use, which improved to about 30% after at least 4 h of practice [8]. Similarly, a hip extension exosuit study reported a 6.2% decrease in metabolic energy in the first session, which improved to a 10.3% reduction by the final session [9]. Such changes are often well described by an exponential relationship between metabolic cost and exposure time, with the time constant varying across individuals and reflecting the rate of adaptation [10]. These findings indicate that user adaptation is a dynamic process distinct from controller tuning, yet it remains less explored in the literature [11]. Therefore, personalized assistance strategies that account for adaptation are critical for maximizing the utility and performance of wearable robots. 

Although user adaptation plays a critical role in determining the efficacy of wearable robots, it remains underexplored relative to personalized assistance strategies that optimize controller parameters or assistance profiles. This imbalance is evident in the literature: only 21.1% of wearable-robot studies report training duration, whereas 31.6% do not mention the adaptation process [11]. As a result, performance is often evaluated under predefined experimental settings or short-term trials that do not capture how walking economy evolves with practice. Consequently, device effects may be under- or overestimated when adaptation is not considered, and inconsistent exposure durations can reduce the reliability of comparative evaluations. Moreover, quantitatively tracking adaptation in real time and incorporating it into a robot control framework remain significant challenges [12]. Therefore, a practical method for quantifying user adaptation during exoskeleton use—ideally without burdensome instrumentation—represents a critical gap this study aims to address. 

A common approach to assessing adaptation is to analyze metabolic energy convergence [13], typically using respiratory gas analysis to measure oxygen consumption and carbon dioxide production. When energy expenditure stabilizes, the user is considered adapted and the assistive force effectively utilized. While physiologically grounded, this approach is challenging to deploy in practice because it requires expensive equipment and can impose physical constraints during experiments. Although portable gas analysis systems have been used to track metabolic changes across repeated training sessions, they still rely on specialized hardware that is not easily integrated into standard robotic control systems [9]. In addition, metabolism-based adaptation assessment often depends on post hoc analysis. For example, Wang et al. developed a regression model based on breathing data [10]. Because the model is trained on the full dataset, convergence and adaptation state cannot be determined reliably during operation and are instead inferred after the experiment. Therefore, real-time adaptation assessment methods that do not rely on conventional metabolic analysis are needed. 

To address these challenges, this study introduces a real-time adaptation estimation framework that relies solely on wearable sensor data, without requiring external metabolic measurement equipment. We hypothesize that as users adapt to exosuit assistance, their gait becomes more consistent, and this stabilization can be captured through IMU-derived gait-variability metrics [14,15]. The primary objective is to develop and validate an LSTM-based model that estimates the user’s adaptation level in real time from gait-variability features during soft hip exosuit-assisted walking. As a secondary objective, we evaluate whether IMU-derived gait-variability metrics track metabolic adaptation. Together, these aims support a practical approach for monitoring user state to inform training protocols and enable adaptation-aware assistance strategies.

## 2. Materials and Methods

### 2.1. Participants

Five healthy male adults (age: 23.42 ± 0.90 years, weight: 73.42 ± 9.56 kg, height: 170.18 ± 2.16 cm) with no history of significant lower-limb injuries and no prior experience with wearable robots participated in this study. Three subjects participated in the data acquisition experiments, and the remaining two participated in the validation experiments. All participants were fully informed about the experimental procedures, and written consent was obtained before participation. The Institutional Review Board of Chung-Ang University reviewed and approved the study protocol.

### 2.2. Equipment

**Figure 1:**
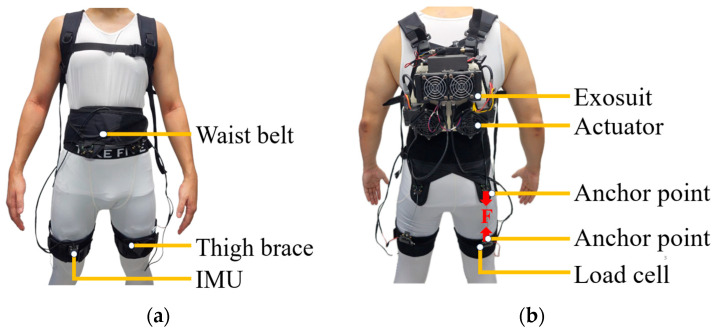
(**a**) Front and (**b**) rear views of the soft hip exosuit. The exosuit comprises a back-mounted actuator module, connected via Bowden cables to thigh braces worn on both legs. The actuator generates an assistive force (F) transmitted to the posterior anchor points of the thigh braces to support hip extension during walking. Load cells positioned on the posterior thighs measure the delivered mechanical force in real time, while inertial measurement units (IMUs) mounted on the anterior thighs record segment orientation and step timing. These IMUs provide the sagittal-plane thigh-angle trajectory used to compute maximum hip flexion and extension during gait. The system integrates textile components (vest, waist belt, and thigh braces) with mechanical and sensing modules to deliver and monitor assistance during locomotion.

The portable soft hip exosuit employed in this study (Figure 1) utilized a cable-driven actuation system that assists only during hip extension, that is, the backward movement of the thigh as the leg pushes off the ground. This movement typically occurs between 30 and 50% of the gait cycle, immediately after the leg reaches its most forward position (maximum hip flexion (MHF)) and before the foot leaves the ground (toe-off). Textile-based elements were integrated with mechanical and control components in this exosuit. The textile system comprised a vest (859 g), waist belt (250 g), two thigh braces (combined weight: 200 g), and a battery pack (183 g).

The mechanical structure included an actuator module (2783 g) housing two actuator units, designed to assist both the left and right hip joints simultaneously. Assistance was delivered via Bowden cables, which transmitted mechanical force from the actuators to the user’s hip joints.

The sensing and control architecture of the soft exosuit integrated multiple components to enable precise real-time assistance during walking. The sensing module comprised two inertial measurement units (IMUs; MTi-630 AHRS, Xsens Technologies B.V., Enschede, Netherlands) and two load cells (LSB205, FUTEK Advanced Sensor Technology, Inc., Irvine, CA, USA). The IMUs were mounted on the anterior side of the thigh braces to estimate the gait cycle percentage (GCP), a critical parameter for timing the assistive force. In this study, the onset of the GCP was defined as the moment of MHF.

Load cells were positioned on the posterior side of the thigh braces to quantify the mechanical interaction between the exosuit and user. These sensors measured the force applied by the exosuit and provided feedback to the control algorithm, enabling it to track the desired assistive force trajectory accurately. Although load cells were used in real time to verify that the exosuit accurately tracked the desired assistive force profile, these data were not analyzed further as outcome measures in this study.

The exosuit was controlled via a CompactRIO system (sbRIO-9651, National Instruments, Austin, TX, USA) and two motor drivers (Gold Twitter, Elmo Motion Control, Petach-Tikva, Israel), implementing an admittance–position switching control scheme [16]. To estimate user adaptation in real-time, a Raspberry Pi 4B+ module was integrated to communicate with the main controller at 800 Hz, processing gait data and transmitting adaptation levels back to the exosuit without disrupting the control loop. Upon receiving real-time data from the exosuit, it continuously evaluated the user’s adaptation level and transmitted the results back to the exosuit.

The experiment was performed on an instrumented treadmill (TM-09-P, Bertec, Columbus, OH, USA). To calculate metabolic costs, a respiratory gas analysis system (K5, Cosmed, Rome, Italy) was used to measure oxygen consumption and carbon dioxide production. Metabolic costs were calculated using the standard Brockway equation [17].

### 2.3. Experiment Protocol

In this study, a systematic data analysis procedure was designed to quantitatively assess the gait-related variables associated with user adaptation. The experiment was conducted over six days, during which participants performed treadmill walking tasks while wearing the soft exosuit under consistent experimental conditions. Because metabolic rate varies across days even within the same individual, a brief no-suit walking condition was included at the start of each experimental day [9]. This trial established a daily metabolic reference, ensuring that metabolic reduction and adaptation analyses were normalized to each day’s baseline rather than confounded by day-to-day physiological fluctuations. The no-suit data were used solely for baseline normalization across the 6-day protocol and were not included in LSTM model training. Throughout each session, participants’ respiratory data were continuously collected in real time to estimate metabolic cost, which was subsequently used to model adaptation curves.

**Figure 2:**
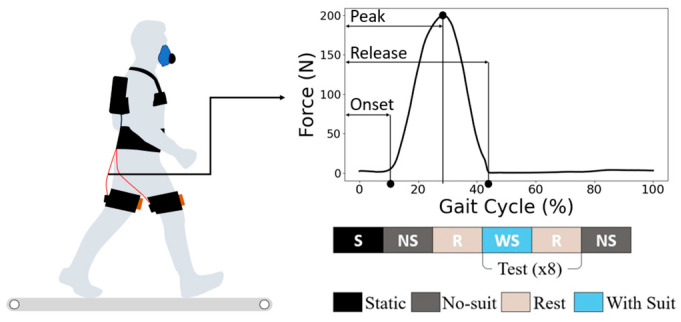
Experiment protocol: Participants walked on a treadmill while wearing the soft exosuit, while the respiratory gas data were collected via a portable metabolic system. The assistive force was applied bilaterally to the hip extensors via a cable-driven mechanism. The assistive force followed a predefined profile with onset, peak, and release timings set at 4, 28, and 42% of the gait cycle, respectively, relative to the timing of maximum hip flexion (MHF). The lower panel illustrates the within-session experimental structure, which consisted of repeated cycles of no-suit (NS), rest (R), and with-suit (WS) walking blocks. Each day’s session began with a static standing (S) baseline measurement to determine resting metabolic rate, followed by eight test cycles. This protocol was repeated over six consecutive days to capture longitudinal adaptation effects.

All participants followed the experimental protocol illustrated in Figure 2. At the beginning of each session, a quiet-standing trial was conducted to determine each participant’s resting metabolic rate. The participants underwent 40 min of exposure daily for 6 days (240 min in total), with a 5 min break included in each session to facilitate fatigue recovery. Each daily session was structured into eight test cycles of alternating no-suit and with-suit walking blocks, interspersed with short rest periods, as shown in Figure 2. All walking trials were performed on an instrumented treadmill set at a constant speed of 1.25 m/s. This speed is commonly used in walking and gait-variability studies and represents a comfortable, energetically neutral pace for most adults [14]. Using a constant speed also minimized speed-related changes in metabolic cost, enabling more precise assessment of adaptation across the six experimental days. 

The assistive force profile was identical for all participants. The onset, peak, and release timings were fixed at 4%, 28%, and 42% of the gait cycle (relative to the MHF event), and the peak force magnitude was kept constant across all users without any individual adjustment. These timings were selected to assist in accordance with the hip-extension moment during level-ground walking [18]. This consistent timing strategy ensured that all the subjects received assistance under the same control parameters during the experiment.

### 2.4. Adaptation Indicators

**Figure 3:**
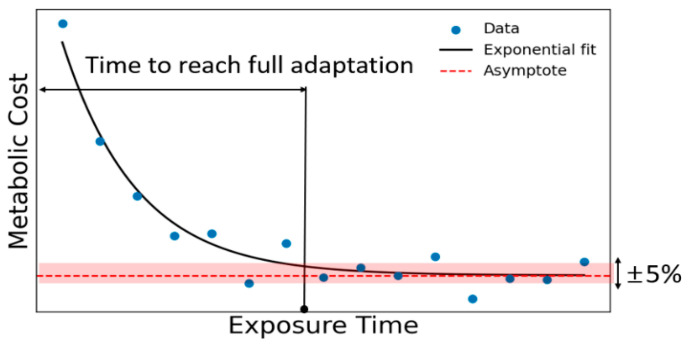
Adaptation graph: Exponential decay of metabolic cost over exposure time. The dashed-red line indicates the asymptotic value of the fitted curve, and the shaded area represents the ±5% threshold.

The metabolic cost of wearable robot users typically decreases exponentially over time, as illustrated in Figure 3. Based on this behavior, adaptation was defined as the point at which the metabolic cost approached within 5% of the asymptotic value of the exponential fit [8]. In this study, we defined the adaptation level as the inverse of this exponential trend, representing the extent to which the metabolic cost approached its steady-state minimum. Accordingly, an adaptation level of 100% was assigned when the metabolic cost reached the asymptotic plateau, providing a continuous, physiologically interpretable ground truth for model training. To evaluate user adaptation during exosuit-assisted walking, we designed a series of experiments and defined a set of gait-based variables, referred to as adaptation indicators. These indicators were selected to capture the characteristic features of locomotor control that may change with repeated exposure to the device. The aim was to establish objective metrics that reflect the user’s adaptation over time. With increasing exposure to the exosuit over multiple days, we examined the evolution of gait variability [14], interpreted as fluctuations in spatiotemporal gait parameters, which indicates the adaptations in locomotor control strategies.

### 2.5. Data Analysis

The analysis of movement variability focused on three key gait parameters: step-frequency variability, MHF angle, and maximum hip-extension angle. Gait variability is not random noise but a physiologically meaningful signature that reflects the state of the neuromotor control system [15]. Stride-to-stride fluctuations exhibit characteristic patterns, beginning with an initial increase during the exploratory phase and progressively decreasing as the locomotor system adapts to new mechanical conditions. Previous studies have demonstrated that variability in gait-related variables initially increases and then decreases as the nervous system adapts, and such changes are associated with reductions in energy cost [14]. Based on these findings, we selected parameters directly related to the function of our device that assist during hip extension, thereby making hip-joint kinematics and step timing particularly relevant to the adaptation process of the user. These parameters were calculated from IMU data attached to the user’s thigh, which recorded 3D inertial measurements at 400 Hz. Before each session, a static upright standing trial was recorded to define the 90° reference for hip flexion. The sagittal-plane thigh angle was then extracted and low-pass filtered at 7 Hz to remove high-frequency sensor noise while preserving the primary kinematic content of human gait. Maximum hip flexion (MHF) and maximum hip extension (MHE) were identified as the peak and trough values within each gait cycle, respectively. Step frequency was computed as the inverse of the stride time, obtained from successive MHF events. The variability of each parameter was quantified as the variance calculated over 10 consecutive gait cycles. Given the within-subject longitudinal design and constant walking speed, we quantified variability using variance rather than the coefficient of variation to focus on adaptation-related changes in absolute step-to-step fluctuations. This analysis was restricted to data from the final 3 min of each session, when participants’ walking was in a steady state. For each session, we computed short-term variability using 10 step windows, then compared values from Days 1 and 6 to assess time-dependent adaptation.

### 2.6. Estimation Model

The LSTM model is a neural network architecture designed to improve predictions for sequential data and has characteristics particularly suited to learning user adaptations that change over time [19]. The hidden and cell states of the LSTM were designed to reflect the wearable data input at each time step, enabling the tracking of adaptation changes over time. Moreover, they could reflect information from previous times in the prediction while remembering it. To predict the adaptation level during exosuit-assisted walking, we employed an LSTM model that generated predictions every 10 steps. The model used three variability parameters as inputs: step-frequency, MHF-angle, and maximum-hip-extension angle variabilities. The model’s output represented the estimated adaptation level. To evaluate predictive accuracy, we considered a prediction accurate if the absolute error was within 10% of the actual value. The tolerance of ±10% was adopted to account for the inherent variability observed in human metabolic measurements. Even under controlled steady-state walking conditions, intra-individual fluctuations of approximately 5% in metabolic cost have been reported [9]. When additional variability introduced by sensor noise, treadmill environment, and day-to-day physiological drift is considered, expanding this margin to ±10% provides a reasonable and physiologically justified threshold for adaptation estimation. Subject-level generalization was assessed using leave-one-subject-out (LOSO) cross-validation across five participants. In each fold, one participant was reserved for testing, while the remaining participants were used for training. Within the training set, a subset was held out for validation to enable early stopping and hyperparameter monitoring. The model was optimized using the mean-squared error loss and trained with the Adam optimizer. The LSTM architecture consisted of recurrent layers with 128 hidden units each, followed by a fully connected output layer. The input features were normalized to zero mean and unit variance before training. The model was trained for 200 epochs with early stopping based on the validation loss.

### 2.7. Statistical Analysis

To statistically evaluate the changes across sessions, we compared the values obtained on Days 1 and 6. A paired *t*-test (α = 0.05) was applied to assess differences in metabolic cost and gait-variability measures. Statistical significance was set at *p* < 0.05.

Although the sample size was small, this parametric test was deemed appropriate given the within-subject design and the robustness of the paired *t*-test to deviations from normality in different scores.

## 3. Results

### 3.1. Metabolic Cost

**Figure 4:**
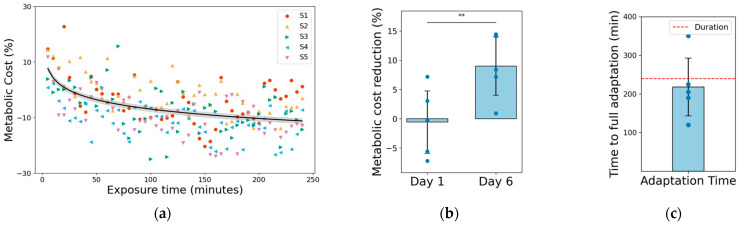
Metabolic cost reduction: (**a**) Metabolic cost (%) over cumulative exposure time. Each marker represents one trial from the five participants (S1–S5), with the black curve denoting the exponential fit and the gray shaded area representing the 95% confidence interval. (**b**) Comparison of metabolic cost reduction between Days 1 and 6. Standard deviation is shown as error bars. The decrease from Day 1 to Day 6 was statistically significant (** indicates paired *t*-test, *p* < 0.01). (**c**) Time required for each participant to reach complete adaptation. The red-dashed line indicates the experimental duration limit (240 min); one participant exceeded this threshold.

Compared with the metabolic cost on Day 1, the cost on Day 6 was reduced by 9.0 ± 5.6% (*p* < 0.01, Figure 4a). This reduction was statistically significant (*p* < 0.01, Figure 4b), validating a clear improvement in walking economy after adaptation. The decline followed an exponential time course; a minimal change was observed during the initial phase, followed by a progressive decrease as the exposure continued. The regression model estimated an average time constant of 202 ± 78 min, with four of five participants adapting within the 240 min experiment, whereas one required approximately 350 min (exceeding the study duration). Notably, this ~10% improvement is consistent with prior multisession training studies [8,9], indicating that our protocol successfully captured the adaptation process reported in an earlier study.

### 3.2. Adaptation Indicators Result

**Figure 5:**
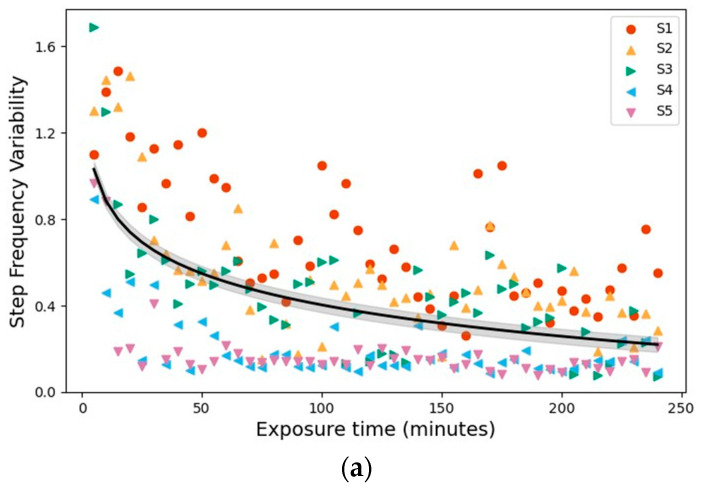

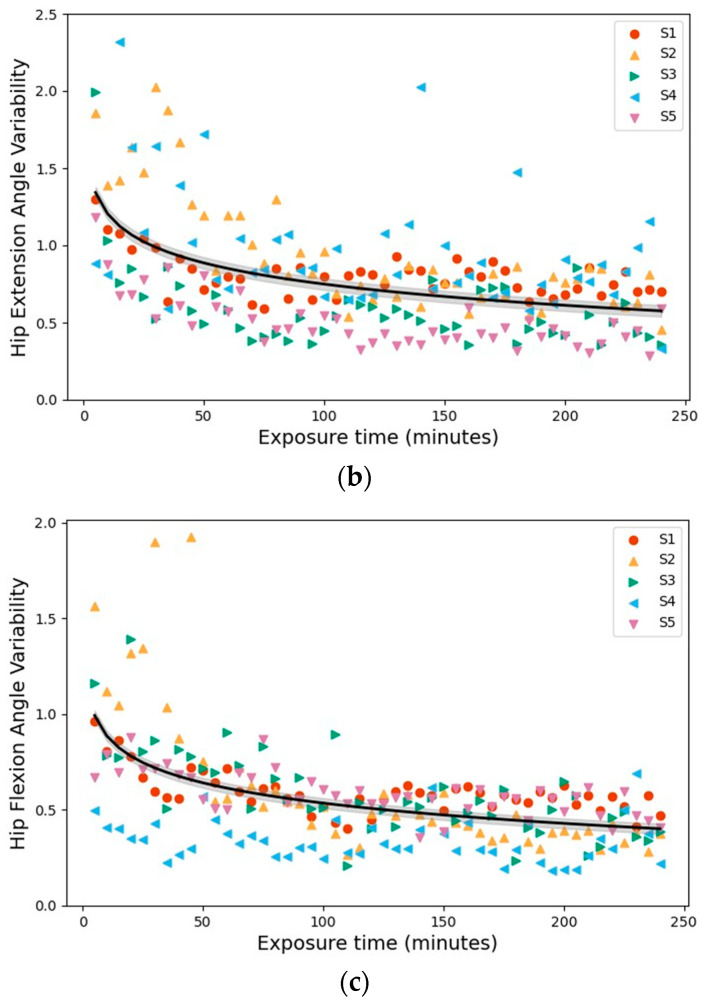
Adaptation Indicators: Changes in gait variability over cumulative exposure time. (**a**) Step-frequency variability. (**b**) Hip-extension-angle variability. (**c**) Hip-flexion-angle variability. Each point represents a trial for each of the five participants. Although the experiments were conducted across six separate days, variability was plotted against cumulative exposure duration to reflect the continuous, time-dependent nature of locomotor adaptation. Black curves denote exponential regression fits with shaded areas indicating the 95% confidence interval.

Over the six days of exosuit-assisted walking, gait variability decreased consistently across all measured parameters, following a time course similar to the decline in metabolic cost. From Days 1 to 6, the step-frequency, hip-flexion-angle, and hip-extension-angle variabilities decreased by 66.4 ± 6.8%, 37.9 ± 24.2%, and 42.8 ± 10.6%, respectively. Among these, the largest adaptation effect was observed in step-frequency variability, which dropped by nearly two-thirds on average, suggesting that the timing consistency and performance markedly improved as the users learned to coordinate with the exosuit. Figure 5 illustrates these trends across subjects, with the exponential regression fits demonstrating the progressive stabilization of gait patterns over repeated exposures.

### 3.3. Level of Adaptation

**Figure 6:**
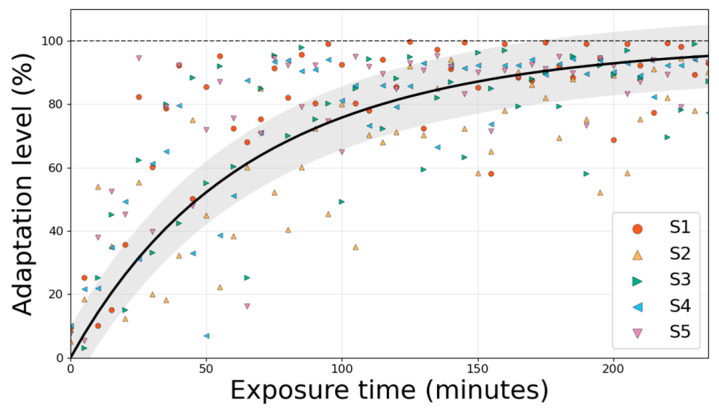
Predicted adaptation level (%) over cumulative exposure time for all five participants under leave-one-subject-out (LOSO) evaluation. Colored markers denote the LSTM-estimated adaptation level for each participant (S1–S5). The black curve indicates the mean metabolic cost–based reference adaptation trajectory (exponential fit) averaged across participants, and the shaded region represents the ±10 percentage-point error band used to define prediction accuracy.

The predicted adaptation level increased rapidly during the initial phase and then plateaued, broadly mirroring the exponential pattern observed in the IMU-derived variability indicators (Figure 6). To strengthen subject-level generalization under the limited sample size, we evaluated the model using leave-one-subject-out (LOSO) cross-validation across all five participants. Across the five LOSO folds, prediction accuracy—defined as the proportion of estimates remaining within ±10 percentage points of the metabolic cost–based adaptation curve—was 58.3–60.4% per subject, with an overall mean of 59.2 ± 1.1%. On average, the model predictions deviated by 12.26 ± 1.27 percentage points (MAE), and the predicted and reference adaptation curves were moderately to strongly correlated (r = 0.79 ± 0.04), with R^2^ = 0.58 ± 0.09. While transient deviations were observed in some subjects, particularly during the early-to-mid adaptation phase, the model consistently captured the later plateau behavior.

Collectively, these results are consistent with the two primary objectives of this study. First, the gait-variability measures derived from wearable IMUs demonstrated consistent changes with repeated exosuit use, paralleling the exponential decline in metabolic costs and supporting their use as real-time indicators of adaptation. Second, the proof-of-concept LSTM model demonstrated subject-level generalization under LOSO evaluation, achieving a prediction accuracy of 59.2% within ±10 percentage points of the metabolic cost–based adaptation curve. The model further demonstrated a mean absolute error (MAE) of 12.26 percentage points and an R^2^ value of 0.58 across unseen subjects. The MAE represents the average absolute deviation between the predicted adaptation level and the metabolic cost-based ground truth across all time windows, indicating an average deviation of approximately 12 percentage points. These findings demonstrate the practicality of the proposed framework and provide a foundation for further refinement.

## 4. Discussion

This study introduced a novel approach to real-time user adaptation evaluation using only wearable sensor data. We observed that the gait-variability metrics, particularly step timing and hip-kinematic variability, consistently decreased over multiple days of exosuit use, closely tracking reductions in metabolic cost as users adapted. An LSTM model leveraging these metrics was able to estimate each user’s adaptation level in real time with moderate accuracy (59.2% of predictions within ±10 percentage points under LOSO evaluation). This emphasis on user adaptation contrasts with most prior exoskeleton studies, which focus on optimizing assistance parameters rather than tracking users’ acclimatization patterns. Our results confirmed that gait variability in step timing and hip angles decreased progressively across repeated sessions, in parallel with reductions in metabolic cost. Step-frequency and hip-angle variabilities emerged as key variables for capturing users’ adjustment to robotic assistance. During normal gait, the neuromuscular system favors energy-efficient and consistent movement patterns [20]. Introducing external assistance initially disrupts this consistency, and the user undergoes an exploratory phase before finding a new stable pattern [14]. The significant reductions observed (e.g., ~66% less step-frequency variability) reflected this exploration of the convergence process. The proposed hip-extension assistance directly influenced hip motion and step timing; therefore, the variability in these measures decreased as the user learned to integrate assistance. Notably, the progressive reduction in gait variability closely mirrored the improvements in metabolic efficiency; as movements became more repeatable and tuned to the exosuit, walking energetics improved. Therefore, these variability metrics are potentially valid real-time indicators of adaptation status, which can inform dynamic adjustments to assistance strategies.

Furthermore, our study demonstrates that an LSTM neural network can be leveraged to estimate a user’s adaptation level from time-series data of these variability metrics. We employed an LSTM model to capture the temporal patterns of adaptation [19]. In our implementation, the LSTM model processed three input features (step-frequency, hip-flexion, and hip-extension variabilities) and output an estimated adaptation level every 10 steps. Because these variability features were extracted from continuous IMU signals in successive 10-step windows, each input segment inherently depended on the preceding gait dynamics. Consequently, the adaptation estimation task exhibited strong temporal dependencies as the user’s gait gradually evolved with repeated exosuit exposure. The recurrent structure of the LSTM enabled the model to retain information across consecutive windows and capture these gradual transitions, making it particularly suitable for real-time prediction of adaptation trends. The network architecture (128 hidden units plus an output layer) provided a balance between modeling capacity and real-time efficiency. This lightweight model can facilitate real-time inference without an excessive computational load. Under leave-one-subject-out (LOSO) evaluation across five participants, the model achieved a prediction performance of 59.2% (i.e., the proportion of estimates within ±10 percentage points of the metabolic cost–based adaptation curve). This corresponded to a mean absolute error of 12.26 percentage points, and the predicted adaptation trajectories showed a moderate correlation with the reference curves (r = 0.79). Notably, a recent data-driven study on ankle exoskeleton users determined that incorporating additional biomechanical inputs improved the prediction of individual gait responses [21], suggesting that including more sensor modalities can further enhance the adaptation estimator.

The observed ~10% improvement in the metabolic cost after approximately 4 h of cumulative exosuit use aligns with prior multisession training studies [8,9], confirming that our protocol induced an adaptation similar to that reported by other researchers. This consistency supports the idea that our variability-based metrics capture genuine adaptation effects rather than experimental noise. For example, a recent study found that providing visual biofeedback can significantly accelerate adaptation to exoskeleton assistance, nearly doubling energy savings in one quarter of a typical training time [22]. These findings highlight the importance of user-focused interventions; therefore, adaptation can be accelerated by encouraging exploration and guiding users toward optimal movement patterns. Our study aimed to quantify adaptation in real time using wearable sensors, moving beyond post hoc analysis toward continuous real-time monitoring. Instead of focusing solely on optimizing the exoskeleton settings, we provide insight into the dynamic evolution of the human-exoskeleton interaction.

However, prediction errors varied across individuals and time, with transient deviations often observed during the early-to-mid adaptation phase while the later plateau was more consistently captured. This indicates room to improve robustness, for example, by incorporating additional features that better represent inter-individual differences or by adopting personalization strategies (e.g., lightweight fine-tuning using a short calibration period). Nevertheless, an adaptation estimator achieving 59.2% accuracy within ±10 percentage points under LOSO evaluation may still be practically useful when integrated into an adaptive control system, where trend tracking and timely detection of plateauing can inform assistance updates. A real-time adaptation metric enables the exoskeleton controller to adjust the assistance dynamically. For instance, an exosuit can begin with minimal support and increase as the user adapts. Integrating the proposed adaptation estimator into an assist-as-needed control framework enables the exoskeleton to personalize support based on the user’s state, aligning with rehabilitation robotics paradigms, where assistance is tuned to the user’s performance [23]. Prior studies have explored adaptive controllers that modulate impedance or assistance based on user intent and performance signals [24]. By incorporating a quantitative adaptation feedback signal, these controllers can make informed decisions, potentially enhancing user comfort and training effectiveness.

Our findings have significant implications for adaptive exoskeletal control and clinical translation. The ability to estimate user adaptation in real time enables wearable robots to implement effective adaptive assistance strategies. Controllers can use the estimated adaptation level as feedback to adjust the assistance in real time, ensuring that the user is neither under- nor over-assisted. In rehabilitation settings, real-time adaptation monitoring can help clinicians tailor training. For example, a therapist can use an adaptation score to decide when to increase task difficulty, thereby motivating patients by showing their progress over time.

### Limitations

All participants in this study were young, healthy adult males; therefore, the observed adaptation patterns may not be generalizable to older individuals or clinical populations. Furthermore, our experiments involved six consecutive days of treadmill walking with a fixed hip-extension assistance profile. Adaptation may follow different trends during long term use, in overground locomotion and stair ambulation, or when using other exosuit designs. Finally, given the extensive six-day protocol, the current study was conducted with a limited sample size; thus, these results should be viewed as a preliminary proof-of-concept demonstrating feasibility rather than a fully generalized model. Additionally, since gait variability is inherently correlated with walking speed, the current constant-speed protocol limits the assessment of speed-dependent effects. Future studies should therefore investigate the model’s robustness under varying speed conditions to ensure its applicability in dynamic, real-world environments.

## 5. Conclusions

In summary, this study introduced a novel method for quantifying user adaptation during hip exosuit-assisted walking using wearable sensor outputs. Over six days of repeated exosuit use, participants showed progressively less step-timing and hip-angle variability, corresponding to an approximate 9% decrease in metabolic cost from the first to last session. An LSTM model trained on these gait-variability features provided real-time estimates of the adaptation level. Under leave-one-subject-out (LOSO) evaluation across five participants, 59.2% of predictions fell within ±10 percentage points of the metabolic cost–based adaptation curve, demonstrating the feasibility of continuously monitoring exoskeleton adaptation without relying on metabolic testing. Although the model’s accuracy was constrained by a small sample size and limited input features, its performance suggests that, with larger, more diverse training data and additional biomechanical inputs, predictive accuracy can be further improved. Overall, our findings introduce a practical approach for real-time adaptation assessment that can be integrated into exosuit control systems. With further development, such adaptive controllers can adjust assistance based on a user’s adaptation state, ultimately improving the usability and effectiveness of wearable robotic assistance in real-world scenarios.

## Data Availability

Data is contained within the article.
